# Regulation of HPV-16 infection by ubiquitination of the L1 major capsid protein

**DOI:** 10.1128/jvi.00285-26

**Published:** 2026-04-13

**Authors:** Oscar Trejo-Cerro, Martina Bergant Marušič, Patryk Wlodarczyk, Urška Repnik, Nika Lovšin, Michael P. Myers, Robert L. Garcea, Lawrence Banks, Justyna Broniarczyk

**Affiliations:** 1 Tumour Virology Laboratory, International Centre for Genetic Engineering and Biotechnology 18469, Trieste, Italy; 2 Sir William Dunn School of Pathology, University of Oxford, Oxford, United Kingdom; 3 Laboratory for Environmental and Life Sciences, University of Nova Gorica 119110, Nova Gorica, Slovenia; 4 Department of Molecular Virology, Adam Mickiewicz University, Poznan, Poland; 5 Central Microscopy, University of Kiel, Kiel, Germany; 6 Faculty of Pharmacy, University of Ljubljana 37663, Ljubljana, Slovenia; 7 BioFrontiers Institute and Department of Molecular, Cellular and Developmental Biology, University of Colorado, Boulder, Colorado, USA; Tufts University School of Medicine, Boston, Massachusetts, USA

**Keywords:** HPV, infection, major capsid protein L1, protein stability, ubiquitination

## Abstract

The L1 major capsid protein of the papillomavirus is critical for both viral assembly and entry into host cells, but the impact of its post-translational modifications on the HPV life cycle remains poorly understood. In this study, we show, for the first time, that HPV16 L1 is ubiquitinated at several conserved lysine residues (K20, K64, K152, K217, K437, K452, and K454) identified by mass spectrometry. Mutations at K64 and K152 prevent the formation of pseudovirions, while other lysine mutants permit the formation of pseudovirions, but these display lower infectivity in several epithelial cell lines. Pre-incubation of HPV-16 pseudovirions with an anti-ubiquitin antibody also significantly reduces their infectivity. Ubiquitination does not appear to be essential for virus attachment, but K452 and K454 are critical for efficient infectious entry, and they impact intracellular trafficking and processing. Taken together, these results demonstrate a critical link between the ubiquitin-conjugating system and HPV capsid proteins and highlight the potential and novel role of L1 protein ubiquitination in regulating different stages of the HPV infection.

**IMPORTANCE:**

Despite vaccination efforts, HPV infection remains a major global health threat. Understanding how the virus interacts with human cells is therefore crucial for the development of new therapeutic strategies. This study reveals a previously unknown role of ubiquitination of the HPV16 L1 capsid protein, which is critical for both virus assembly and infectious entry. The Ub-acceptor sites identified by mass spectrometry are highly conserved between different papillomavirus types (HPV-16 and BPV-1), indicating a conserved function within the L1 protein and the viral capsid. We show that L1 ubiquitination plays important but diverse roles during HPV infection, potentially influencing both correct intracellular virus trafficking and HPV virion assembly. Overall, these studies demonstrate a critical link between the ubiquitin-conjugating system and HPV capsid proteins and highlight the novel role of ubiquitination during HPV infection.

## INTRODUCTION

Human papillomaviruses (HPVs) are small, non-enveloped DNA viruses. Over 200 different HPV types have been identified, most of which cause benign epithelial lesions. However, a small subset of HPVs is known to cause cancer and is responsible for almost 5% of all human malignancies. These carcinogenic HPVs primarily cause tumors in the cervix, other anogenital regions, and the head and neck (1–3). Although prophylactic vaccines have been introduced to prevent HPV infections, the development of additional antiviral strategies for existing and new infections, particularly in low- and middle-income countries, is still urgently needed.

Papillomaviruses (PVs) have an 8-kb DNA genome enclosed in an icosahedral capsid consisting of 360 copies of the major capsid protein L1 and 12–36 copies of the minor capsid protein L2. Both L1 and L2 are required for capsid assembly and virus entry (4, 5).

The process of HPV entry into the host cells involves several steps, including attachment to the extracellular matrix, structural modifications of the virion, receptor binding, endocytic uptake, trafficking through the endosomal compartment, uncoating, and recruitment of the host cell transport machinery to ensure delivery of the viral genome into the nucleus via the trans-Golgi network and possibly via the endoplasmic reticulum (6–9). The entry of the L2/viral DNA complex into the nucleus is also dependent on mitosis (10, 11).

The L1 protein of HPV-16 is crucial for the assembly of the virus capsid and is the major capsid protein that forms the protective shell around the viral genome. It consists of several domains, including the N-terminal arm, a core region with eight β-strands that form a β-barrel structure, and the C-terminal arm. The N-terminal part facilitates the interactions during capsid assembly and stabilizes the interactions between L1 monomers. The core region forms a β-barrel scaffold that provides stability, with loops and turns contributing to the capsid architecture. The C-terminal part stabilizes the capsid structure and can interact with host cell factors during viral entry (12–14).

In the core region, five hypervariable L1 surface loops were identified, ranging from 10 to 30 amino acids in length. These loops, referred to as BC (AA 50–69), DE (AA 110–153), EF (AA 160–189), FG (AA 262–291), and HI loops (AA 348–360), are also targeted by human antibodies during the immune response. These loops are crucial for capsid formation. Amino acid changes in these regions might affect immune recognition and impair the structural integrity of the L1 protein and the efficient assembly of viral particles (12–15).

Ubiquitination (Ub) is a highly dynamic enzymatic process in which an 8 kDa ubiquitin molecule is covalently bound to a corresponding lysine (K) residue on the target protein. This process involves several enzymatic reactions to transfer ubiquitin from the E1 ubiquitin-activating enzyme to the E2 ubiquitin-conjugating enzyme and finally to the E3 ubiquitin-protein ligase, which transfers the ubiquitin moiety to the target protein (16).

A broad spectrum of viruses from different viral classes, including RNA and DNA viruses, has been shown to utilize ubiquitin. These viruses employ Ub at different stages of their life cycle, using both ubiquitination and deubiquitination processes (17–22). The early viral proteins of human papillomaviruses have developed complex interactions with the ubiquitin-conjugating system (23, 24), including the well-known role of the E6-E6AP complex, which mediates ubiquitination and degradation of host cell p53 (25).

Interestingly, very little is known about the role of ubiquitination of HPV capsid proteins in the different stages of the HPV life cycle. Therefore, we were interested in understanding the link between the ubiquitin-conjugative system and HPV L1 major capsid protein, which could give us insights into the HPV infection cycle.

To address this question, we performed mass spectrometry analysis to identify potential conserved ubiquitin-binding sites in the L1 proteins of HPV-16 and bovine papillomavirus 1 (BPV-1). We used purified HPV-16 and BPV-1 pseudovirions (PsVs) produced in cultured cells and compared them with native purified BPV particles isolated from bovine papillomas. As a result, we identified several highly conserved ubiquitin-acceptor sites on both HPV-16 and BPV-1 L1 proteins. Interestingly, some mutations of certain Ub acceptor sites block PsV assembly, while others significantly inhibit the viral infectious entry process and impair the capsid disassembly process.

## RESULTS

### Identification of conserved Ub-acceptor sites in HPV-16 L1

**Fig 1:**
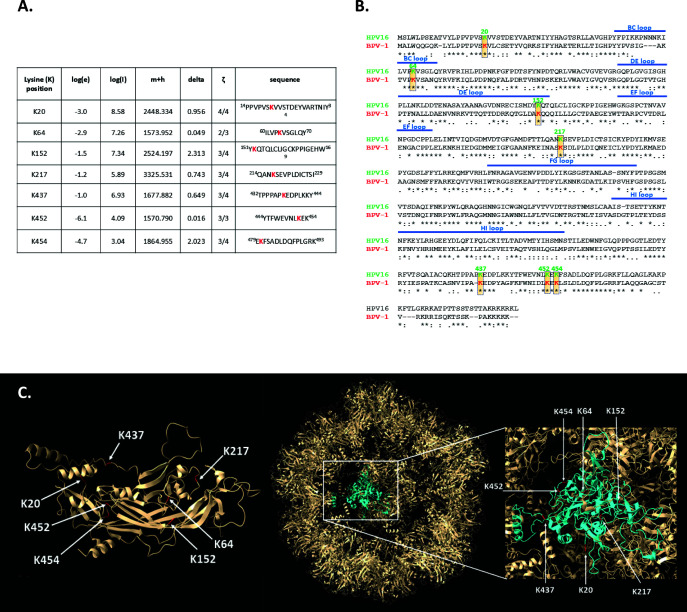
Identification of conserved Ub-acceptor sites in the HPV-16 L1 capsid protein using mass spectrometry. (**A**) The bicistronic plasmids expressing HPV-16 L1 and L2 were transfected into HEK293TT cells. After 48 h, the cells were harvested, and pseudovirions were purified by cesium chloride gradient and analyzed by mass spectrometry to identify Ub-modifications. The lysine residues K20, K64, K152, K217, K437, K452, and K454 were found ubiquitinated in HPV-16 PsVs. The table shows results from the mass spectrometric analysis: log(e) is the base 10 log of the expectation that the assignment is stochastic; log(l) is the base 10 log of the sum of the intensities of the fragment ion spectra; m+h is the calculated mass of the protonated parent ion for this sequence assignment; delta is the difference between the measured and calculated protonated parent ion masses; and ζ is the ratio of the measured charge of the parent ion to the number of basic sites in the assigned peptide sequence. The sequence of the assigned peptide is also shown. (**B**) Alignment of L1 protein sequences of HPV-16 and BPV-1. Alignment was generated with ClustalW software. Conserved lysine Ub-acceptor sites in the L1 sequences are highlighted in yellow and boxed. (**C**) The positions of the identified Ub-acceptor sites displayed on the 3D model of HPV-16 L1 as a monomer (left panel) or the T=1 particle of HPV-16 L1 (right panel) were presented using Chimera X software.

To determine whether the papillomavirus capsid proteins are Ub-modified and whether these modifications are found within intact capsids, we performed a mass spectrometry analysis of HPV-16 PsVs produced in HEK293TT cells. Several ubiquitin modifications of L1 were identified, including lysine residues K20, K64, K152, K217, K437, K452, and K454 (Fig. 1A and C). Interestingly, all of the identified Ub-acceptor sites are highly conserved between HPV-16 (an alpha-papillomavirus) and BPV-1 (a delta-papillomavirus) (Fig. 1B).

### Confirmation of HPV-16 L1 ubiquitination

**Fig 2:**
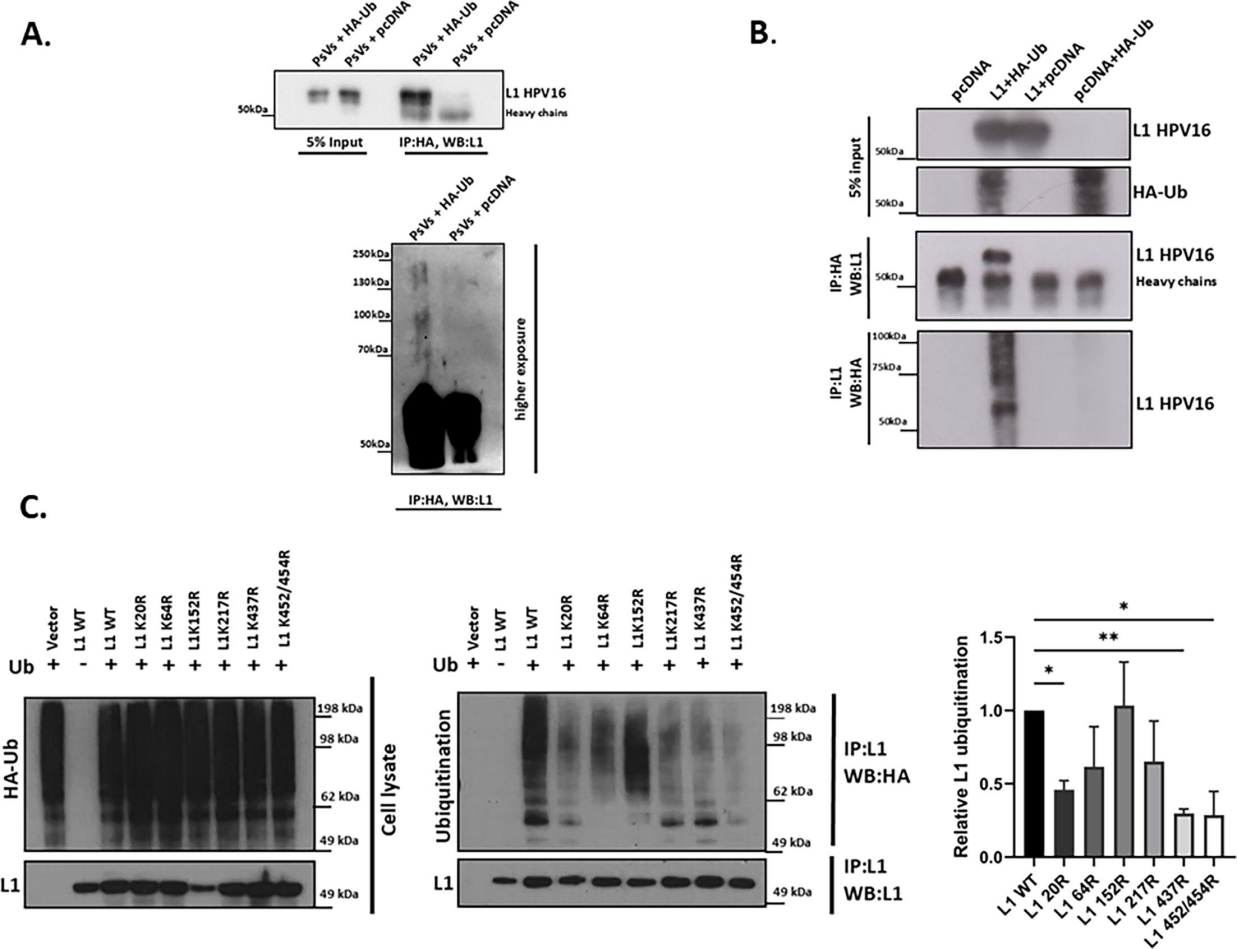
Confirmation of L1 ubiquitination. (**A**) HPV-16 wild-type PsVs were produced in HEK293TT cells in the presence of UbHA or pcDNA plasmids. CsCl gradient-purified PsVs+UbHA and PsVs+pcDNA were immunoprecipitated with HA-agarose beads and analyzed by western blotting using the anti-L1 HPV-16 antibody. The lower panel shows a higher-exposure image from an independent ubiquitination assay. (**B**) HEK293 cells were transfected with the following plasmids: pcDNA alone, HPV-16 L1 plus HA-ubiquitin, HPV-16 L1 plus pcDNA, and pcDNA plus HA-ubiquitin. After 24 h, half the cell extract was immunoprecipitated using anti-HA antibody-conjugated agarose beads, while the second half was incubated with anti-L1 antibody. The ubiquitinated L1 was then detected by western blotting using an anti-HPV-16 L1 antibody or an anti-HA antibody, respectively. The upper panels show HPV-16 L1 and Ub inputs. (**C**) HEK293 cells were transfected with the pcDNA and HA-ubiquitin, wild-type HPV-16 L1 alone as a control and HPV-16 L1 mutants (K20R, K64R, K152R, K217R, K437R, and K452R/K454R) plus HA-ubiquitin. After 24 h, the cell extracts were immunoprecipitated using anti-L1 antibody-conjugated agarose beads. The ubiquitinated L1 was detected by western blotting using an anti-HA antibody. The left panel shows representative images of the western blot analyses. The right panel shows quantification of L1 ubiquitination, normalized to amounts of immunoprecipitated L1. The results are expressed as the means from at least three independent experiments, and the standard deviations are shown (*P < 0.05; **P < 0.01).

To further prove that ubiquitin is incorporated into HPV-16 PsVs, the bicistronic plasmid expressing HPV-16 L1 and L2 was transfected into HEK293TT cells, together with HA-ubiquitin (UbHA) or pcDNA plasmids as a control. After 48 h, the cells were harvested, and pseudovirions were purified by CsCl gradient. Purified PsVs+UbHA and PsVs+pcDNA were immunoprecipitated using HA-agarose beads and analyzed by western blotting using the anti- HPV-16 L1 antibody. As shown in Fig. 2A, HA-ubiquitin was incorporated into HPV-16 PsVs during virus production, attached to the L1 protein. Moreover, L1 ubiquitination in purified PsVs, produced under standard conditions in HEK293TT cells in the absence of HA-Ub overexpression, was also demonstrated by immunoprecipitation with an anti-ubiquitin antibody (data not shown).

Next, to confirm the L1 ubiquitination, HEK293TT cells were transfected with pcDNA plasmid alone as a control, or with plasmids expressing HPV-16 L1 and HA-ubiquitin, either separately or in combination. After 24 h, the cell extracts were divided: the first part was immunoprecipitated using anti-HA antibody-conjugated agarose beads, while the second was incubated with an anti-L1 antibody and protein G-conjugated Sepharose beads. The ubiquitinated L1 was then detected by western blotting using an anti-L1 HPV-16 antibody or an anti-HA antibody, respectively. As shown in Fig. 2B, there was a clear ubiquitination of HPV-16 L1 in the HEK293TT cells. To verify further that all Ub acceptor sites identified by mass spectrometry in the L1 sequence can bind ubiquitin, we generated several HPV-16 L1 mutants (K20R, K64R, K152R, K217R, K437R, and K452R/K454R). HEK293 cells were transfected either with pcDNA or with plasmids expressing HA-ubiquitin, together with plasmids expressing the HPV-16 L1 wild-type or HPV-16 L1 mutants. After 24 h, the cell extracts were immunoprecipitated using anti-L1 antibody-conjugated agarose beads. The ubiquitinated L1 was then detected by western blotting using an anti-HA antibody. The results shown in Fig. 2C confirm the presence of L1 ubiquitination, which is decreased upon introducing the mutation (substituting lysine with arginine, K>R) in the predicted Ub acceptor sites. It also appears that not all identified lysines contribute equally to the overall ubiquitination of L1, as the substitution of different lysines by arginines reduces the ubiquitination of L1 to different degrees.

### Mutations of the HPV-16 L1 Ub-acceptor sites do not alter L1 stability

Having shown that L1 is ubiquitinated at several lysine residues, we next wanted to determine how mutations at Ub-acceptor sites might translate into changes in L1 protein levels and whether L1 stability can be regulated by proteasomal or lysosomal degradation. To do this, we used the plasmids expressing HPV-16 L1 wild-type or the K>R mutations (K20R, K64R, K152R, K217R, K437R, and K452R/K454R). We also generated mutations in HPV-16 L1 Ub acceptor sites in the context of the bicistronic plasmid expressing both L1 and L2 of HPV-16. HEK293TT cells were transfected with plasmids expressing the wild-type and the mutated (K20R, K64R, K152R, K217R, K437R, and K452R/K454R) L1 alone or L1 and L2 together. After transfection, cells were treated with the proteasome inhibitor CBZ or left untreated. To confirm that CBZ inhibitors worked, we used cells transfected with plasmids expressing HPV-16 E7, as it is well-known that treatment with CBZ inhibits the degradation of ectopically expressed HPV E7 oncoproteins (26).

**Fig 3:**
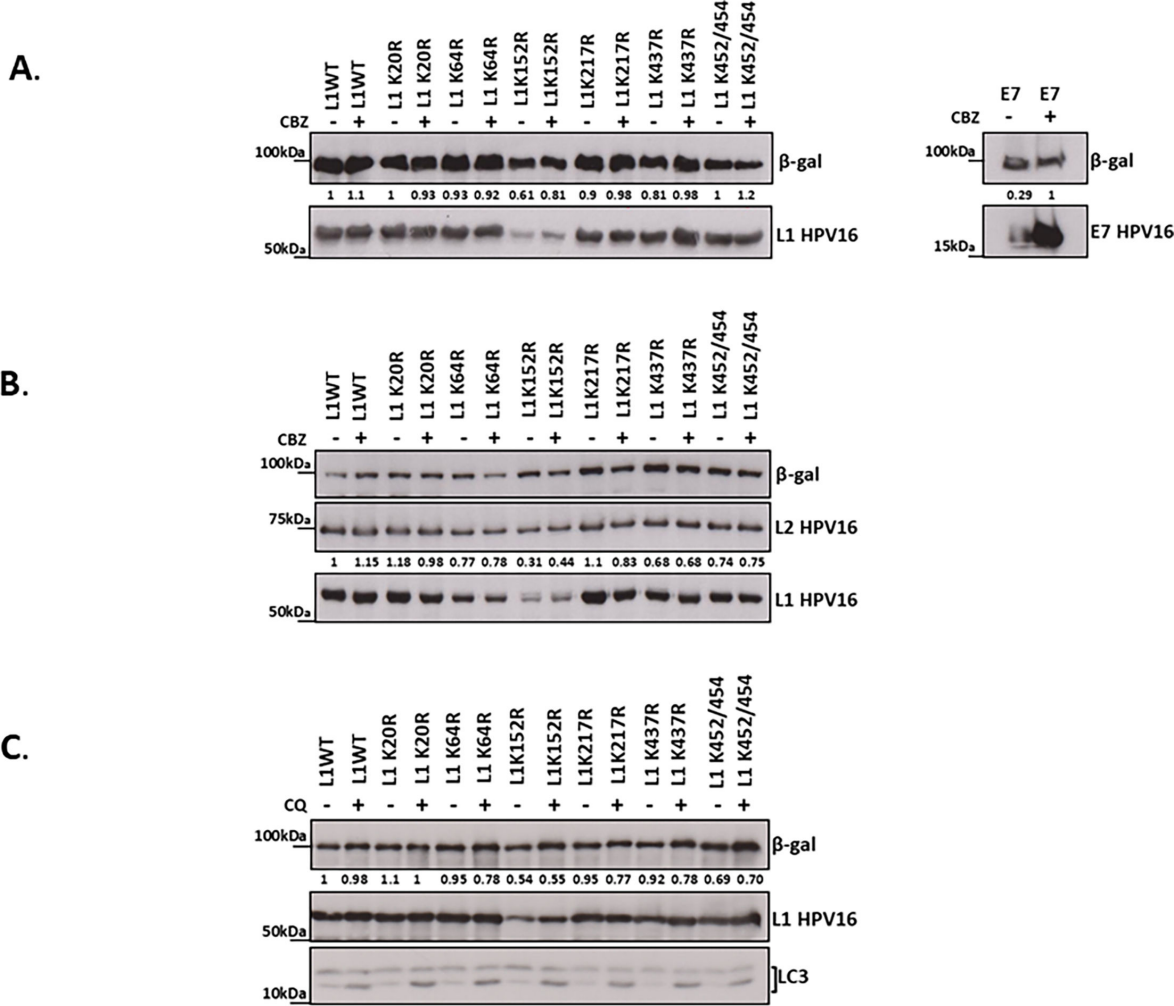
Mutation of the HPV-16 L1 Ub-acceptor sites has no major effect on L1 stability. (**A**) HEK293TT cells were transfected with plasmids expressing the wild-type and the mutated L1, as indicated, and 100 ng of lacZ plasmid, as a control to account for transfection efficiency. After 24 h, the cells were incubated with or without proteasome inhibitor CBZ, harvested 24 h later, and analyzed by western blotting, using anti-HPV-16 L1 and anti-β-galactosidase antibodies. β-Gal was used as the loading and transfection efficiency control. As a CBZ-positive control, cells were transfected with plasmids expressing HPV-16 E7 and anti-HPV16 E7 antibody and 100 ng of lacZ plasmid, as a control to account for transfection efficiency. (**B**) HEK293TT cells were transfected with psheLL plasmids expressing HPV-16 L1 and L2, containing the wild-type or the mutant L1, as indicated, and 100 ng of lacZ plasmid as a control to account for transfection efficiency. After 24 h, the cells were incubated with or without proteasome inhibitor CBZ and harvested 24 h later. Cell lysates were collected and analyzed by western blotting, using anti-HPV-16 L1, anti-HPV-16 L2, and anti-β-galactosidase antibodies. (**C**) HEK293TT cells were transfected with plasmids expressing wild-type or mutated L1, and 100 ng of lacZ plasmid as a control to account for transfection efficiency. After 24 h, the cells were incubated with or without chloroquine and harvested 24 h later. Cell lysates were collected and analyzed by western blotting, using anti-HPV-16 L1, anti-β-galactosidase antibody, and anti-LC3 antibody as chloroquine treatment-positive control. For all experiments, the L1 level was assessed by quantifying the pixel intensity of the L1 band relative to the corresponding intensity of the β-galactosidase band, using densitometry (ImageJ). The results are shown normalized to the levels of L1 wild-type untreated control.

Next, the levels of L1 and E7 were analyzed by western blotting. Interestingly, as shown in Fig. 3A and B, only the K152R substitution decreased the levels of L1 to any degree (40%), while other mutations in Ub-acceptor sites had only modest effects on L1 protein levels. As expected, the presence of L2 did not affect L1 stability (Fig. 3B). Treatment with the CBZ proteasome inhibitor drastically increased E7 stability (70%), confirming that the inhibitor worked well but did not lead to a dramatic increase in L1 K152R protein level, suggesting that L1 K152R protein does not undergo proteasome-dependent degradation (Fig. 3A and B).

To determine whether L1 stability might be regulated by lysosomal degradation, we performed a similar experiment in which HEK293 cells were transfected with plasmids expressing either HPV-16 L1 wild-type alone or with the HPV-16 L1 mutants. We then blocked lysosomal degradation with chloroquine, which resulted in only a minor increase in L1 K152R protein levels, indicating that L1 K152R levels are also not regulated by the lysosome. The chloroquine activity was demonstrated by the accumulation of the lysosomal protein LAMP5, which was detected with the LC3 antibody (Fig. 3C). Based on these results, we conclude that the main role of HPV-16 L1 ubiquitination is not to regulate the stability of the L1 capsid protein. The unaltered levels of L1 K152R protein upon the inhibition of proteasomal and lysosomal pathways suggest that its degradation might be driven by an intrinsic instability rather than by ubiquitin-mediated mechanisms.

### Analysis of the role of mutations in HPV-16 L1 ubiquitin acceptor sites in PsV assembly and stability

**Fig 4:**
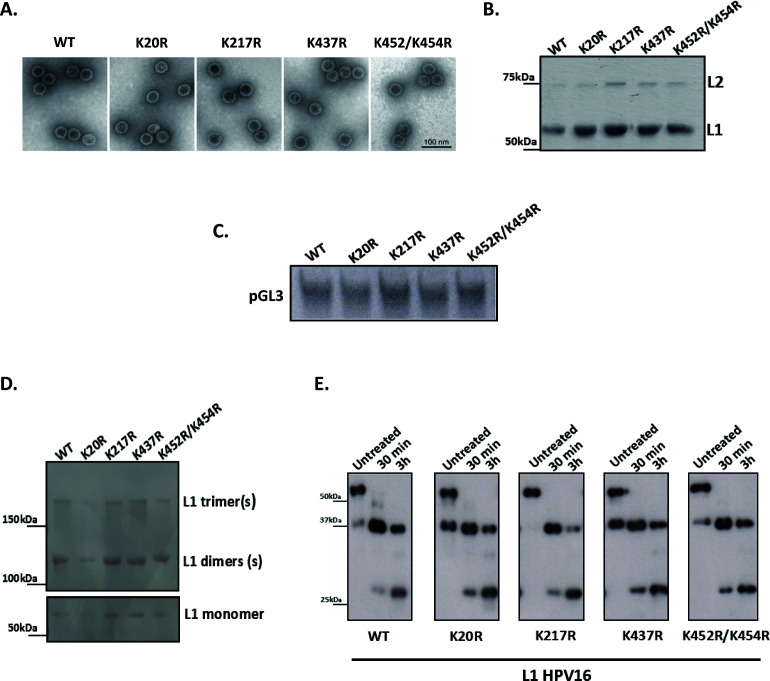
Analysis of the role of mutations in HPV-16 L1 ubiquitin acceptor sites in PsV assembly and stability. (**A**) Negative-staining transmission electron microscopy of purified wild-type and mutant PsVs showed no structural differences. Note that the mutated K64R and K152R PsVs failed in production. (**B**) Coomassie-stained gels of the synthesized and purified PsVs (K20R, K217R, K437R, and K452R/K454R) show similar levels of capsid protein for wild-type and mutated PsVs, indicating that co-assembly of L1/L2 proteins into particles is unaffected by the K>R substitution. (**C**) The agarose gel shows similar amounts of encapsidated DNA (pGL3 vector carrying the luciferase reporter gene) extracted from the assembled and purified wild-type and mutant PsVs. (**D**) The patterns of disulfide bonds in the assembled and purified wild-type and mutated HPV-16 PsVs were examined by nonreducing denaturing gel analysis and Ponceau S staining (upper panel). The lower panel shows conventional denaturing gel analysis of all PsV preparations. Note that all PsV preparations show similar levels of disulfide cross-linking, which correspond to those of fully mature capsids. (**E**) The stability of wild-type and mutated HPV-16 PsVs was verified by trypsin digestion. Purified wild-type and mutated HPV-16 PsVs were incubated with an equal volume of 0.05% trypsin at pH 7.0 for either 30 min or 3 h and compared with untreated samples by western blotting analysis. Note that mutations of the L1 Ub-acceptor sites do not alter the PsV stability.

We next sought to determine whether L1 ubiquitination is important for PsV assembly. HEK293TT cells were transfected with bicistronic plasmids expressing the wild-type or the mutated (K20R, K64R, K152R, K217R, K437R, and K452R/K454R) L1 and L2 proteins, together with a luciferase reporter plasmid (pGL3). After 48 h, the cells were harvested, and the wild-type (WT) and mutant PsVs were purified using CsCl gradient and imaged by negative-staining transmission electron microscopy (EM). Interestingly, not only K152R but also K64R substitutions blocked PsV production (data not shown), while no major structural differences were observed between the wild-type and the other mutant PsV preparations (K20R, K217R, K437R, and K452R/K454R) (Fig. 4A).

To determine whether the loss of the Ub-acceptor sites in HPV-16 L1 can affect the co-assembly of L1/L2 proteins into particles, purified wild-type and mutated PsVs (K20R, K217R, K437R, and K452R/K454R) were resolved by 10% SDS-PAGE. Coomassie-stained gels (Fig. 4B) show similar ratios of L1–L2 within the capsid. Additionally, agarose gel electrophoresis of the reporter vector containing luciferase (pGL3), extracted from purified wild-type and mutated PsVs (K20R, K217R, K437R, and K452R/K454R), showed that they packaged similar amounts of pGL3 plasmid (Fig. 4C), which was confirmed by real-time PCR analysis. The average number of pseudogenome (pGL3 plasmid) copies incorporated per 1 mg of L1, in at least three independent experiments, was similar for wild-type and all mutated PsV preparations and consistent with the number of pseudogenome copies incorporated per 1 mg of L1 (2.0E 11 copies/mg L1) observed previously in HEK293TT cells (27).

It is well known that capsid maturation depends on intermolecular disulfide bonds that enhance the stability of papillomavirus capsids (28). To determine if there are differences in disulfide cross-linking levels between wild-type and mutated PsV samples, we examined them using nonreducing denaturing gels. As shown in Fig. 4D, HPV-16 wild-type and the mutated virion samples display similar patterns of L1 dimers and trimers, indicating fully mature capsids, as previously reported (29).

Next, we explored whether the K20R, K217R, K437R, and K452R/K454R mutations might affect capsid stability. Previous studies have shown that short trypsin treatments have little effect on mature and correctly folded HPV-16 PsVs (30, 31). To determine whether there might be slight conformational differences between the wild-type and mutant HPV-16 PsVs that could be detected by trypsin sensitivity, we conducted trypsin digestions of PsV samples for 30 min or 3 h (Fig. 4E). Detection of L1 after trypsin digestion showed similar tryptic products in most of the PsV preparations, indicating that the mutations in these ubiquitin acceptor sites in the HPV-16 L1 have little or no effect on the virion sensitivity to trypsin.

Taken together, these data indicate that mutations of certain Ub-acceptor sites in the L1 sequence (K64R, K152R) block HPV-16 PsV production, while mutations at other Ub-acceptor sites (K20R, K217R, K437R, and K452R/K454R) do not affect PsV assembly and stability.

### The Ub antibody significantly reduces HPV-16 infection

**Fig 5:**
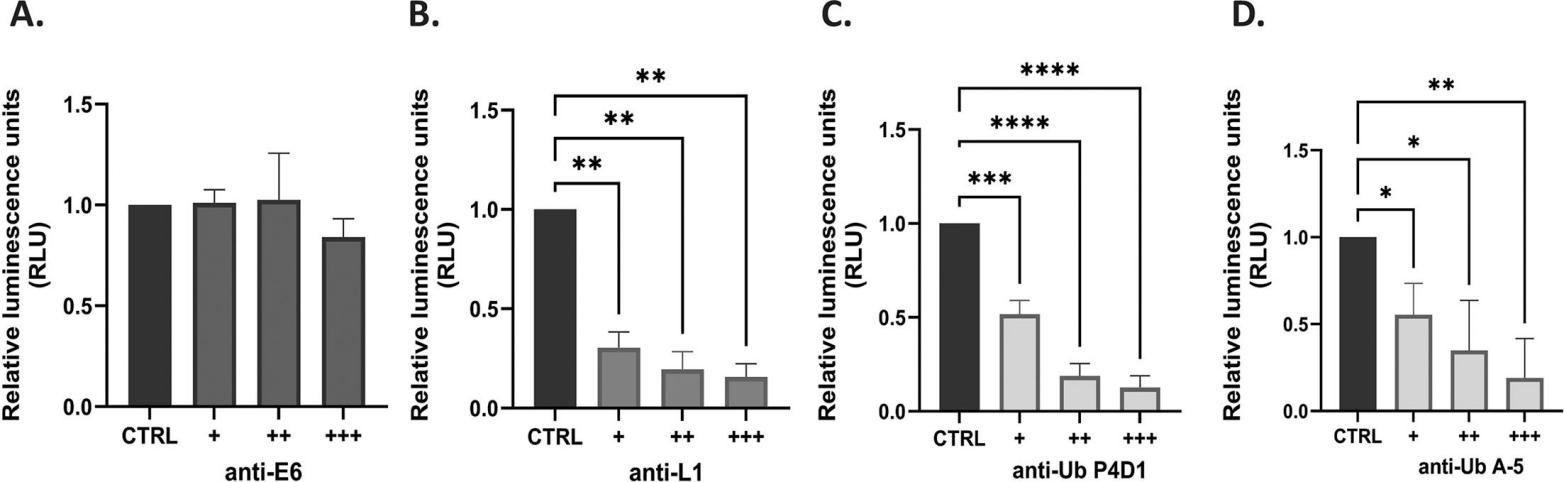
The Ub antibody significantly reduces HPV-16 infection. Wild-type HPV-16 PsVs were incubated with increased concentration of control anti-HPV-18 E6 (control) (**A**), neutralizing anti-L1 (H16.V5) (**B**), and anti-ubiquitin antibodies P4D1 (**C**) and A-5 (**D**) for 2 h at 37°C and then used to infect HaCaT cells. After 48 h, the cells were harvested, and luciferase activity was measured in triplicate by luminometry. The infection efficiency of antibody-treated HPV-16 PsVs was calculated by normalizing the values to HPV-16 PsVs treated with no antibodies (CTRL). Throughout the experiments, equal amounts of total cell protein extract were used in the luciferase measurements. The results are expressed as means ± SD of at least three independent experiments (*P < 0.05; **P < 0.01, ***P < 0.001, ****P < 0.0001).

To test whether the ubiquitin antibody can bind to the intact PsVs and impair their infectivity, HPV-16 wild-type PsVs were incubated with increased concentrations of anti-18 E6 (control), neutralizing anti-L1 (H16.V5), and two different anti-ubiquitin antibodies (P4D1 and A-5) for 2 h at 37°C and then used to infect HaCaT cells. After 48 h, the cells were harvested, and the luciferase activity was measured. Interestingly, both anti-ubiquitin antibodies significantly reduced HPV-16 infection in a dose-dependent manner, as shown in Fig. 5C and D. A similar effect was observed when HPV-16 PsVs were incubated with the neutralizing L1 antibody (Fig. 5B), while the non-specific anti-18 E6 antibody did not affect HPV-16 infection (Fig. 5A). All these data suggest that ubiquitin plays an important role in the HPV infectious entry process and that some of the Ub-acceptor sites are exposed on the surface of PsVs.

### Mutations in L1 Ub-acceptor sites reduce the infectivity of HPV-16 PsVs

**Fig 6:**
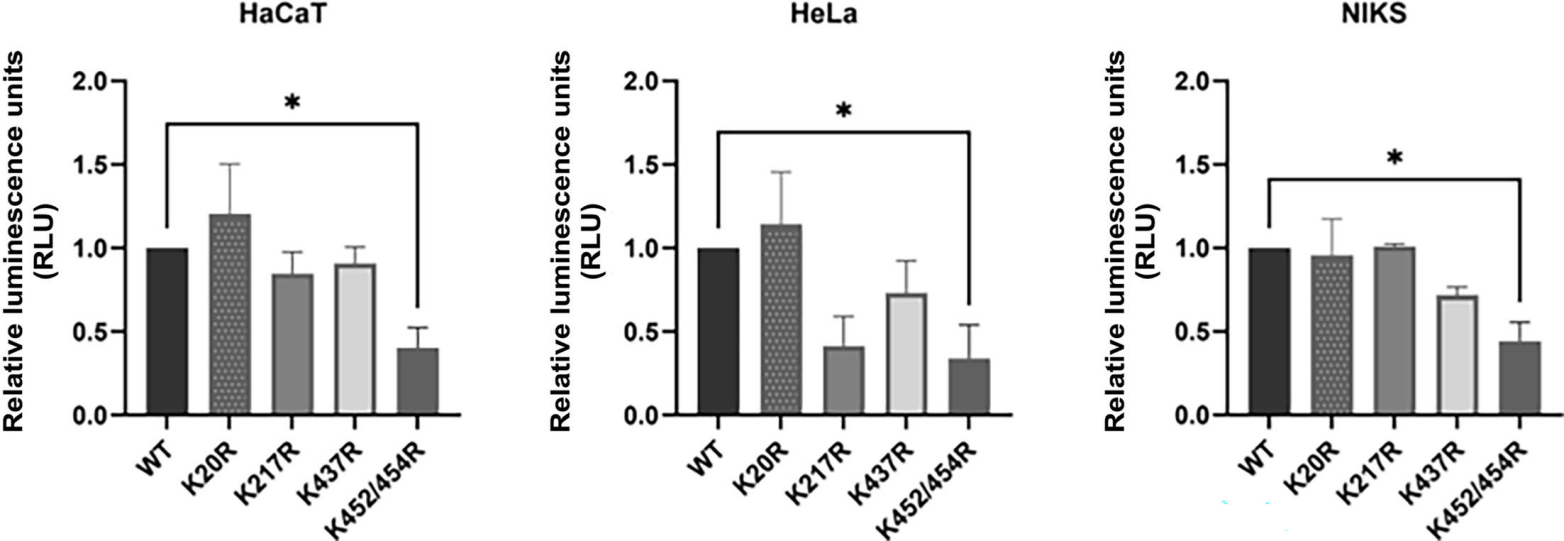
Mutations in L1 Ub-acceptor sites reduce infection with HPV-16 PsVs. HaCaT, HeLa, and NIKS cells were infected with the wild-type (WT) or mutated (K20R, K217R, K437R, and K452R/K454R) HPV-16 PsVs encapsidating equal amounts of the luciferase reporter plasmid. After 36 h, the cells were harvested, and luciferase activity was measured in triplicate by luminometry. The infection efficiency of mutated HPV-16 PsVs was calculated by normalizing the values to wild-type HPV-16 PsVs. Throughout the experiments, equal amounts of total cell protein extract were used in the luciferase measurements. The results are expressed as means ± SD of at least three independent experiments (*P < 0.05).

We have shown that mutations in some L1 Ub-acceptor sites (K20R, K217R, K437R, and K452R/K454R) do not affect PsV assembly and stability and that anti-ubiquitin antibodies impair the general HPV-16 PsV infectivity. Next, we wanted to determine which of these residues might be required for virus infectivity by comparing the abilities of the wild-type and the mutant HPV-16 PsVs (K20R, K217R, K437R, and K452R/K454R) to transduce a luciferase reporter construct. Infections were performed in HaCaT, HeLa, and NIKs cell lines, and luciferase activity was measured in cells harvested at 36 h post-infection (hpi). As shown in Fig. 6, mutations in L1 Ub-acceptor sites generally reduced the ability of HPV-16 PsVs to infect these cell lines to varying degrees in the different cell lines. The strongest inhibition was observed with the K452R/K454R substitution in HPV-16 L1, which had significant inhibitory effects (over 50%) on the ability of HPV-16 to infect all cell lines tested. Taken together, these results suggest that intact Ub-acceptor sites in L1, especially K452R/K454R, play an important role in HPV-16 infection in different cell lines.

### TAK-243 inhibits HPV16 PsV infectivity and L1 ubiquitination

To independently confirm the importance of L1 ubiquitination during infection, we produced HPV-16 PsVs in the presence of the TAK-243 inhibitor, which blocks ubiquitin-activating enzymes (E1), inhibits ubiquitin conjugation, and disrupts both monoubiquitin signaling and global protein ubiquitination (32).

**Fig 7:**
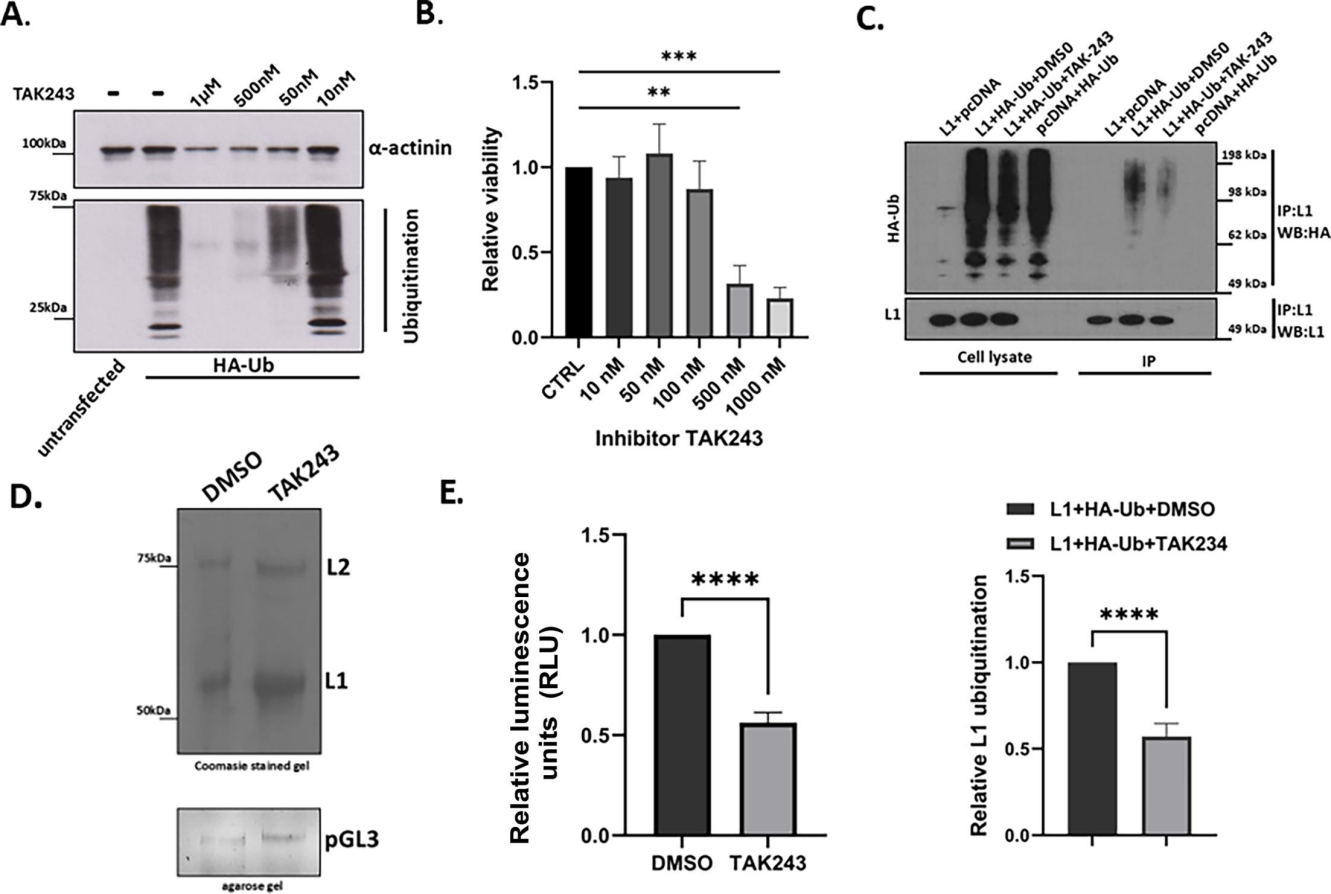
TAK-243 inhibitor reduces HPV16 PsV infectivity and L1 ubiquitination. (**A**) HEK293TT cells were transfected with equal amounts (500 ng) of the HA-Ub plasmid. Five hours after transfection, cells were treated with TAK-243 at varying concentrations (10, 50, 500, and 1,000 nM). After 48 h of treatment, the cells were collected, and total protein extracts were analyzed by western blotting using an anti-HA antibody and anti-α-actinin was used as a loading control. (**B**) HEK293TT cells were exposed for 48 h to increasing concentrations of the TAK-243 inhibitor (10, 50, 100, 500, and 1,000 nM), with DMSO serving as the vehicle control. Cell viability was assessed using the PreMix WST-1 Cell Proliferation Assay System. For each experiment, cell numbers in TAK-243–treated samples were normalized to those in DMSO-treated control cells (CTRL). Bar graphs represent the ratio of viable cells, expressed as the mean of at least three independent experiments, with error bars indicating standard deviation (**P < 0.01, ***P < 0.001). (**C**) HEK293TT cells were transfected with the following plasmid combinations: HPV-16 L1 plus pcDNA, HPV-16 L1 plus HA-ubiquitin, or pcDNA plus HA-ubiquitin; 24 h post-transfection, the cells expressing HPV-16 L1 and HA-ubiquitin were treated with either DMSO or the TAK-243 inhibitor (50 nM). After 48 h, cell lysates were subjected to immunoprecipitation using anti-L1 antibody–conjugated agarose beads. Ubiquitinated L1 was detected by western blotting with an anti-HA antibody. Representative western blot images are shown in the upper panel, while the lower panel displays quantification of L1 ubiquitination normalized to the amount of immunoprecipitated L1. Data are presented as mean ± SD from at least three independent experiments (****P < 0.0001). (**D**) HEK293TT cells were transfected with psheLL plasmids encoding wild-type HPV16 L1 and L2; 24 h post-transfection, cells were treated with either DMSO or TAK-243 (50 nM) and harvested after 48 h. The expression of L1 and L2 proteins and the presence of encapsidated DNA were subsequently assessed using Coomassie-stained SDS–PAGE gels (upper panel) and agarose gel electrophoresis (lower panel), respectively. (**E**) HaCaT cells were infected with HPV-16 pseudoviruses (PsVs) generated in the presence of either DMSO or the TAK-243 inhibitor (50 nM), each encapsidating equivalent amounts of a luciferase reporter plasmid. Cells were harvested 36 h post-infection, and luciferase activity was quantified in triplicate using luminometry. Infection efficiency of mutant HPV-16 PsVs was determined by normalizing luciferase values to those obtained with wild-type HPV-16 PsVs. Equal amounts of total cellular protein were used for all luciferase assays. Data are presented as mean ± SD from at least three independent experiments (****P < 0.0001).

First, to verify that the TAK-243 inhibitor reduces protein ubiquitination, HEK293TT cells were transfected with equal amounts (500 ng) of the HA-Ub plasmid; 24 h post-transfection, cells were treated with increasing concentrations of TAK-243 (10 nM, 50 nM, 500 nM, and 1,000 nM). After 24 h of treatment, cells were harvested, and total protein lysates were analyzed by western blot using anti-HA antibodies, with anti-α-actinin serving as a loading control. As shown in Fig. 7A, TAK-243 treatment at 50 nM, 500 nM, and 1,000 nM markedly decreased the ubiquitination pattern compared with untreated cells, whereas no detectable effect was observed at 10 nM. At the same time, we evaluated the effect of TAK-243 on cell viability. HEK293TT cells were exposed to a similar range of concentrations for 24 h, with DMSO as the vehicle control. As shown in Fig. 7B, TAK-243 at 500 and 1,000 nM markedly reduced cell viability, whereas 10, 50, and 100 nM did not result in significant cytotoxicity.

To confirm that TAK-243 specifically also affects L1 ubiquitination, HEK293TT cells were transfected with the following plasmid combinations: HPV16 L1 plus pcDNA, HPV16 L1 plus HA-ubiquitin, or pcDNA plus HA-ubiquitin; 24 h post-transfection, cells expressing HPV16 L1 and HA-ubiquitin were treated with either DMSO or TAK-243 (50 nM). At 48 h post-transfection, cell lysates were immunoprecipitated with an anti-L1 antibody and protein G–Sepharose beads. Ubiquitinated L1 was subsequently detected by western blotting with an anti-HA antibody. As shown in Fig. 7C, treatment with 50 nM TAK-243 resulted in a marked reduction in HPV16 L1 ubiquitination in HEK293TT cells.

Since TAK-243 at 50 nM did not significantly affect HEK293TT cell viability but markedly reduced cellular and L1 ubiquitination signals, this concentration was selected for the PsV production. HEK293TT cells were transfected with psheLL plasmids encoding wild-type HPV16 L1 and L2; 24 h post-transfection, cells were treated with either DMSO or TAK-243 (50 nM) and harvested 24 h later. To assess whether TAK-243 affected L1/L2 co-assembly into viral particles, purified HPV16 PsVs produced in the presence of DMSO or TAK-243 were analyzed by 10% SDS-PAGE. Coomassie blue staining (Fig. 7D, upper panel) showed the presence of L1 and L2 in capsids under both conditions. In addition, agarose gel electrophoresis of the luciferase reporter plasmid (pGL3) extracted from purified PsVs produced with DMSO or TAK-243 demonstrated successful packaging of the pGL3 genome (Fig. 7D, lower panel), which was further quantified by real-time PCR analysis.

To assess whether TAK-243 affects viral infectivity, we compared the abilities of HPV16 PsVs produced in the presence of either DMSO or TAK-243 to transduce a luciferase reporter. Infections were performed in HaCaT cells, and luciferase activity was measured at 36 hpi. As shown in Fig. 7E, PsVs generated in the presence of TAK-243 exhibited a significant reduction in infectivity, with luciferase activity decreased by more than 50% compared with the DMSO control.

### Mutations of the HPV-16 L1 Ub-acceptor sites do not affect HPV binding to the cell surface or early internalization

To better understand the potential function of L1 ubiquitination in infectious HPV entry, we investigated how mutation of the K452R/K454R mutant, which significantly reduced HPV-16 infection, might affect this process.

**Fig 8:**
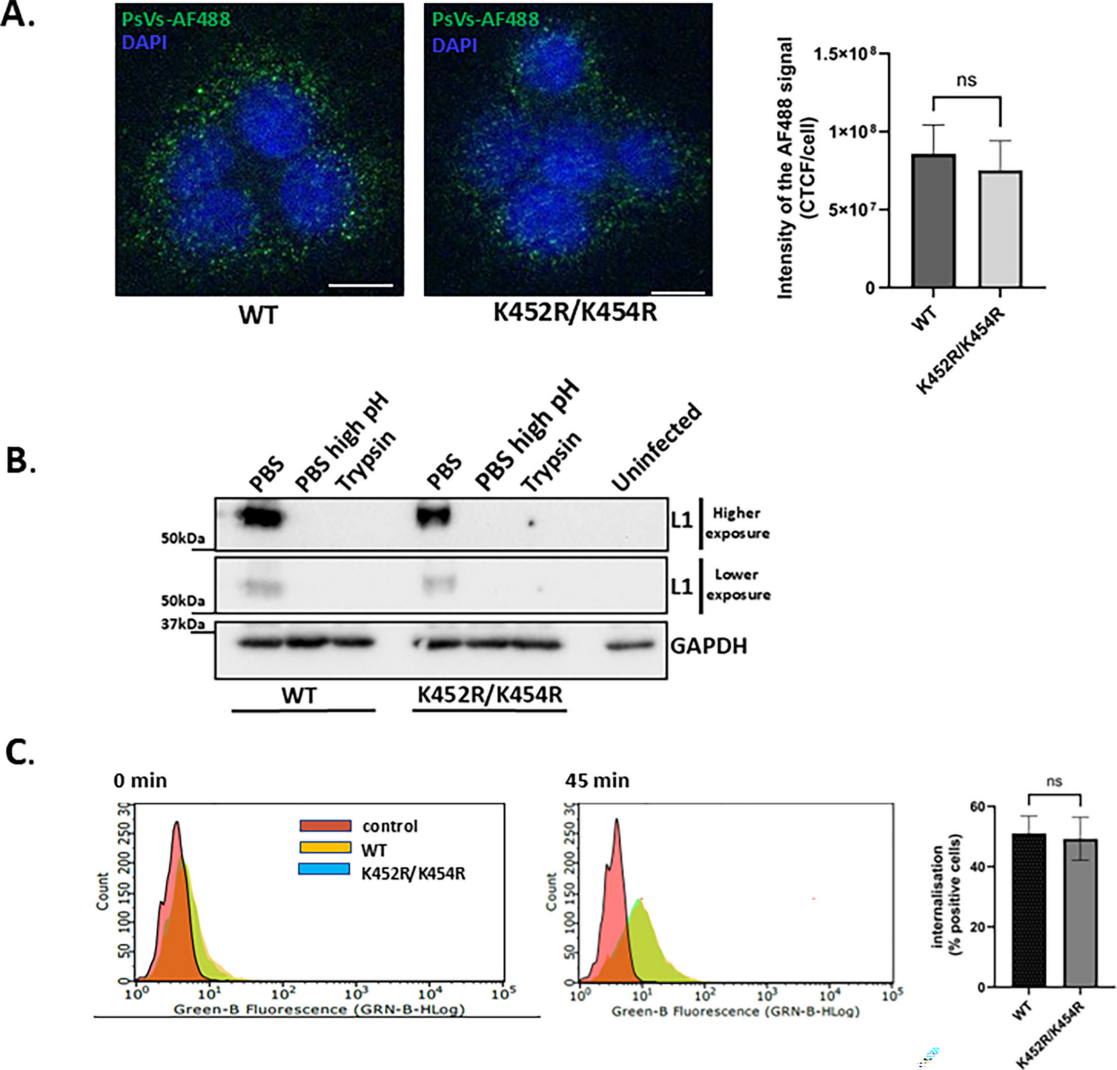
Mutations of the HPV-16 L1 Ub-acceptor sites do not affect HPV binding to the cell surface. (**A**) HaCaT cells were exposed to AlexaFluor 488 (AF488)-conjugated wild-type or K452R/K454R HPV-16 PsVs for 1 h at 4°C. The cells were then washed, fixed, and subjected to quantitative immunofluorescence analysis of nonpermeabilized cells. Representative images show AF488-conjugated PsVs (green) and DAPI-stained nuclei (blue). Scale bar = 10 µm. The intensity of the L1 signal was analyzed by ImageJ and presented as relative corrected total cell fluorescence (CTCF) per cell. (**B**) HaCaT cells were exposed to wild-type or K452R/K454R HPV-16 PsVs for 1h at 4°C. Cells were then washed with regular PBS to remove unbound PsVs or with high-pH PBS to remove the surface-bound PsVs. Cell surface-bound L1 was detected by non-reducing SDS-PAGE and western blotting, using an anti-HPV-16 L1 antibody. (**C**) Flow cytometry analysis of HaCaT cells infected with AF488-conjugated wild-type and K452R/K454R HPV-16 PsVs. The representative histogram plots after 0 and 45 min of internalization are shown compared with the control non-infected cells. AF488-positive cells were quantified using quadrant statistical analysis. All results are expressed as means ± SD of at least three independent experiments (ns, non-significant).

First, to determine whether the K452R/K454R substitutions affect PsV binding to the cell surface, HaCaT cells were incubated with wild-type or K452R/K454R HPV-16 PsVs conjugated with AlexaFluor 488 (AF488) for 1 h at 4°C. Cells were then washed, fixed, and subjected to quantitative immunofluorescence analysis of non-permeabilized cells. As shown in Fig. 8A, similar patterns of L1 staining were observed with both wild-type and mutant PsVs (*P* = 0.2385), indicating that similar quantities of the two PsVs were bound to the cells.

To further confirm this, a similar assay was performed in which the total PsV protein content bound to the cell surface was determined. HaCaT cells were incubated with wild-type or K452R/K454R HPV-16 PsVs for 1 h at 4°C. Cells were then washed with regular PBS to remove the unbound virus or with high-pH PBS to also remove the surface-bound PsVs. Cell surface-bound L1 was detected by non-reducing SDS-PAGE and western blotting, using an anti-L1 HPV-16 antibody. The data presented in Fig. 8B clearly show that mutation of K452/K454R has no significant impact on virion binding and that high pH washing removes nearly all surface-bound virus, which is important for the entry assays described below. After confirming that attachment is not impaired by altered ubiquitination of L1 at positions K452 and K454, we analyzed internalization of PsVs 45 min post-attachment. HaCaT cells were incubated with AF488-conjugated wild-type or K452R/K454R HPV-16 PsVs, as described above. After binding, the cells were washed, and the bound PsVs were allowed to internalize at 37°C for 45 min. Cells were then treated with trypsin to detach them and remove the surface-bound PsVs. Upon analysis by flow cytometry, a similar number of positive cells was seen in both cases (51% for wild-type and 49% for K452R/K454R) (Fig. 8C), indicating that neither cell attachment nor early internalization is affected by altered L1 ubiquitination.

### Altered L1 ubiquitination impairs PsV processing and intracellular trafficking

**Fig 9:**
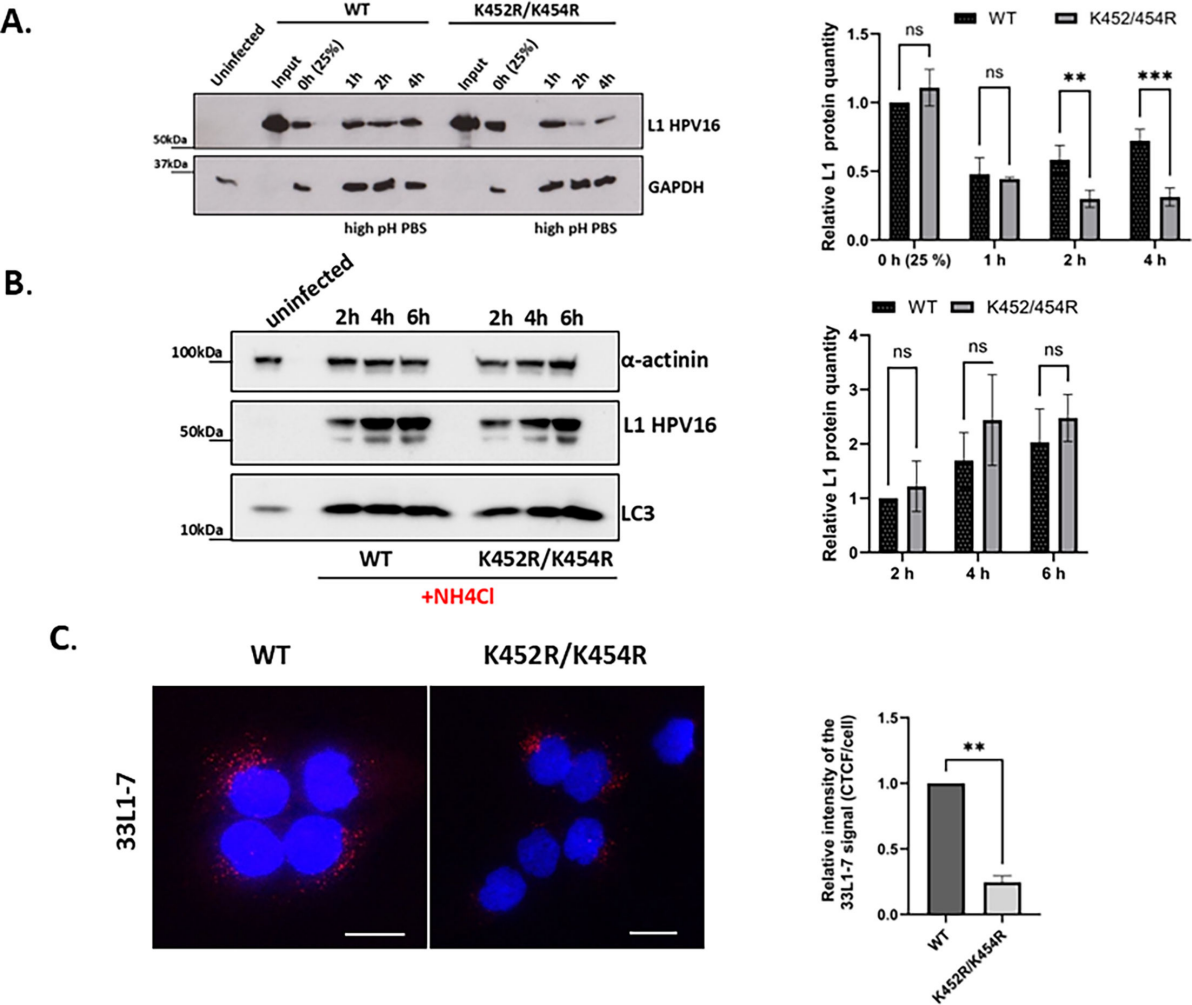
Mutations of the HPV-16 L1 Ub-acceptor sites affect HPV intracellular processing. HaCaT cells were left untreated (**A**) or were treated prior to infection with the acidification inhibitor NH_4_Cl (**B**). Cells were then infected with wild-type or K452R/K454R HPV-16 PsVs. At different times post-infection, non-internalized viruses were removed by high-pH PBS wash. Cells were then harvested, and the levels of L1 protein remaining were determined by western blotting. L1 levels were assessed by quantifying the pixel intensity of the L1 bands relative to the corresponding intensity of the GAPDH bands using densitometry. The results are normalized to the level of wild-type L1 after attachment (0 h) (**A**) or 2 h post-infection (**B**). LC3 antibody was used as a positive control for acidification. The representative blots and cumulative densitometry results are shown. (**C**) HaCaT cells were exposed to nonconjugated wild-type or K452R/K454R HPV-16 PsVs for 1 h at 4°C. Cells were then washed and incubated at 37°C for 6 h. After incubation, cells were fixed and stained for L1 protein using 33L1-7 antibody. Selected images show uncoating-dependent L1 signal (33L1-7 antibody) in red and DAPI-stained nuclei in blue. Scale bar = 10 µm. The intensity of the L1 signal was analyzed by ImageJ and presented as relative corrected total cell fluorescence (CTCF) per cell. All the micrographs are representative and show the mid-cell body/nucleus focal planes. All results are expressed as means ± SD of at least three independent experiments (**P < 0.01; ***P < 0.001; ns, nonsignificant).

To determine at what point altered L1 ubiquitination affects HPV intracellular processing, HaCaT cells were incubated with wild-type or K452R/K454R HPV-16 PsVs for 1 h at 4°C to allow the virions to attach to the cell membrane (0 h). At different times after attachment (1 h, 2 h, and 4 h), the cells were treated with high-pH PBS to remove any non-internalised virus and then harvested. The levels of L1 protein attached and internalized into the cells were determined by western blotting. The results in Fig. 9A show similar levels of L1 in wild-type and K452R/K454R PsVs after 1 h incubation at 4°C (0 h), confirming that there are no major differences in the attachment efficiency of wild-type or mutant PsVs. No differences in L1 levels were observed 1 h after attachment, suggesting that mutations of the HPV-16 L1 Ub-acceptor sites do not affect the early internalization step, as shown already in Fig. 8C. While efficiencies of early internalization appeared to be very similar, the amount of K452R/K454R L1 detected was much lower than that of wild-type L1 at the later time points (2 and 4 h), suggesting that the intracellular processing of HPV-16 PsVs in HaCaT cells is affected by altered L1 ubiquitination.

To determine whether the lower amount of K452R/K454R L1 is a consequence of lysosomal degradation, the assay was repeated in the presence of the lysosome inhibitor ammonium chloride. As can be seen from Fig. 9A and B, clear rescue of L1 is seen in the presence of ammonium chloride (2, 4, and 6 h post-infection), indicating that mutations of the HPV-16 L1 Ub-acceptor sites do not delay the PsV internalization but rather increase the rate of PsV degradation.

We then sought to determine whether the rapid degradation of K452R/K454R HPV-16 affects the HPV-16 capsid disassembly at 6 hpi, which is required for effective transition of the L2-DNA complex to the nucleus (33).

To monitor viral uncoating, HaCaT cells were infected with wild-type and K452R/K454R PsVs and stained with the 33L1-7 antibody, which recognizes L1 only after partial disassembly of the viral capsid. We observed significantly lower uncoating-dependent 33L1-7 signal in cells infected with mutant K452R/K454R PsVs compared with wild-type HPV-16 PsVs 6 hpi (*P*= 0.0021) (Fig. 9C). The staining for the 33L1-7 uncoating marker was normalized to the total L1 signal per cell (data not shown), thereby correcting for differences in overall abundance of L1. The reduced 33L1-7 staining thus reflects a specific reduction in uncoating at 6 h post-infection relative to the whole L1 internalized pool, rather than a consequence of a lower amount of total L1 protein at this time point.

**Fig 10:**
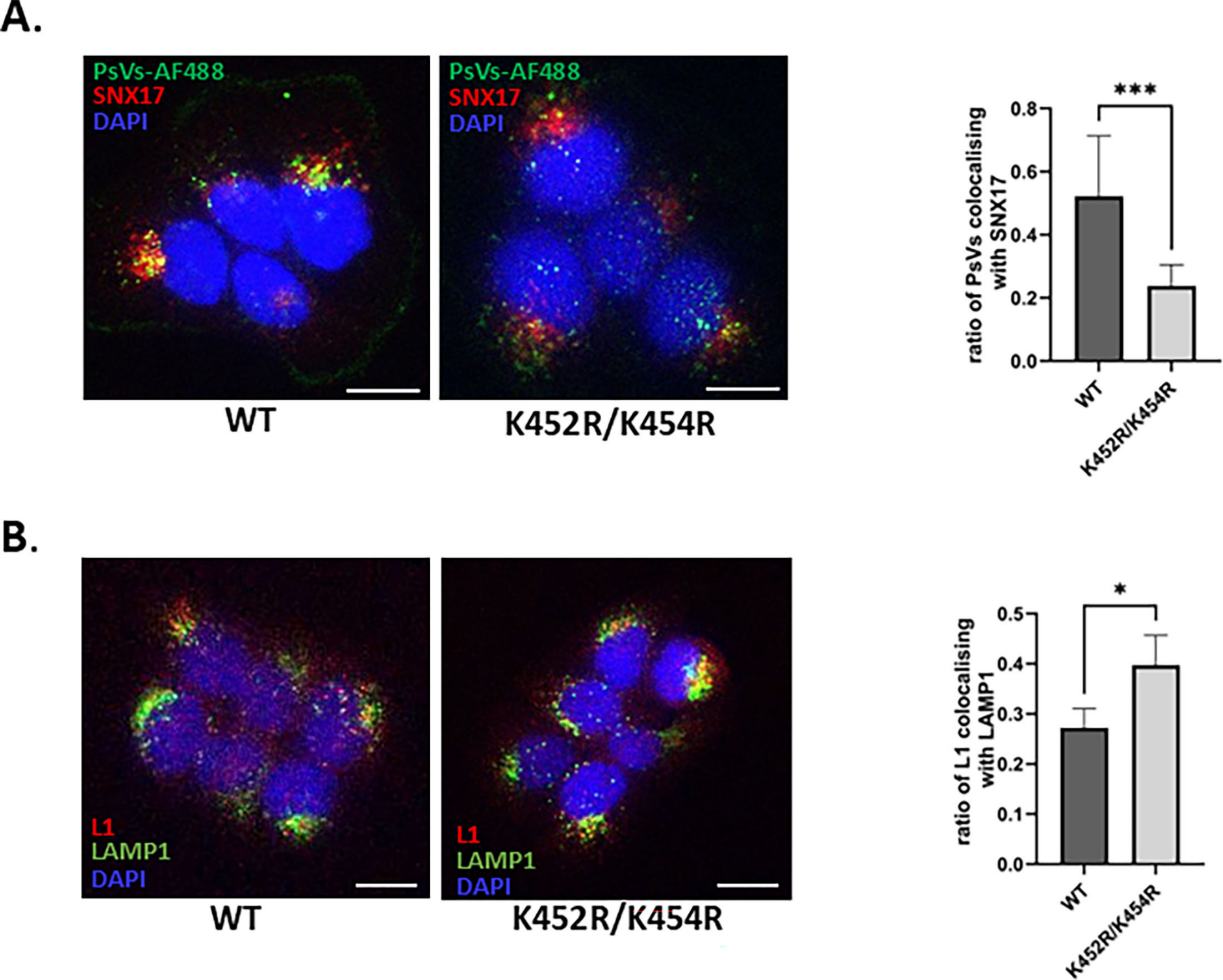
Mutations of the HPV L1 Ub-acceptor sites impair early HPV-16 intracellular trafficking. HaCaT cells were exposed to AF488-conjugated (**A**) or nonconjugated (**B**) wild-type or K452R/K454R HPV-16 PsVs for 1 h at 4°C. Cells were then washed and incubated at 37°C for the indicated times. After incubation, cells were fixed and stained for SNX17, LAMP1, and L1 protein. (**A**) Selected images of HPV-16 PsV-SNX17 colocalization at 2 h post-infection. Graphs show the ratios of PsVs colocalizing with SNX17 to the SNX17-positive vesicles colocalizing with PsVs. (**B**) Selected images of HPV-16 L1-LAMP1 colocalization at 6 h post-infection. The graph shows the ratio of L1 colocalizing with LAMP1-positive vesicles to LAMP1-positive vesicles colocalizing with L1. All the micrographs are representative and show the mid-cell body/nucleus focal planes. Scale bar = 10 µm. Ratios of colocalization were determined with Mander’s colocalization coefficients for two channels, using ImageJ software with the JACoP plug-in. The results from three independent experiments are presented as means ± SD, and the corresponding *P* values are *P < 0.05; ***P < 0.001.

To determine whether altered intracellular processing can interfere with the intracellular trafficking of K452R/K454R HPV-16 capsids, we performed a series of immunofluorescence experiments. First, we analyzed their colocalization with the host sorting nexin-17 (SNX17) protein, an established milestone of the early intracellular trafficking of HPV-16. SNX17 interacts with the HPV L2 protein and controls the trafficking of the L2–DNA complexes (34). We infected HaCaT cells with AF488-conjugated wild-type and K452R/K454R HPV-16 PsVs and analyzed their colocalization with SNX17-positive vesicles 2 hpi. We found that the ratio of wild-type PsVs colocalizing with SNX17-positive vesicles (Fig. 10A) was very similar to the data from our previous study (35). In contrast, K452R/K454R PsVs showed a significantly lower ratio of colocalization (*P* = 0.0008) with the SNX17-positive vesicles at 2 hpi.

Based on the reduced amounts of full-length L1 observed later during internalization (Fig. 9A), the rapid passage of K452R/K454R PsVs through the endocytic compartments could be responsible for the low colocalization of PsVs with SNX17-positive vesicles at 2 hpi and the fast degradation of L1 later. Interestingly, the level of L1 protein colocalizing with the lysosomal marker LAMP1 was significantly higher in mutant K452R/K454R PsVs, compared with wild-type PsVs (*P* = 0.0119) (Fig. 10B), suggesting that ubiquitin-deficient PsVs are transported to the lysosomal compartments at a higher rate.

Taken together, these data suggest that ubiquitination of the HPV-16 L1 protein plays an important role during HPV-16 infection, leading to rapid L1 degradation and impairment of normal intracellular trafficking, which is subsequently reflected in the overall infectivity of HPV-16 PsVs.

## DISCUSSION

Several viruses can exploit the ubiquitin system to facilitate their successful infection (17–21). In the case of HPV, the oncoproteins E6 and E7 can interact with several E3 ubiquitin-protein ligases, leading to the degradation of their targets in host cells (23, 36). In addition to the well-known role of ubiquitination in labeling proteins for degradation, ubiquitination is involved in the regulation of many biological processes, such as lysosomal protein degradation, cell division, autophagy, endocytosis, trafficking, and cell signaling (37–39).

Using mass spectrometry analysis and biochemical assays, we showed that HPV-16 L1 PsVs are ubiquitinated at several lysine residues. Ubiquitination and other ubiquitin-like modifiers, such as sumoylation, have already been reported on HPV16 L2 minor capsid proteins (40–42). Although ISGylation of HPV-16 L1 (43) has also been shown, this is the first study that reports a potential functional role for ubiquitination of HPV-16 L1 protein. We generated L1 mutants in which the ubiquitin acceptor sites were disrupted and then investigated the effects of these mutations on virion assembly and infection. We were able to show that the viral capsid protein L1 of HPV can be ubiquitinated and incorporated into infectious virions. Moreover, ubiquitination of L1 appears to be required for efficient viral infection.

Mass spectrometry analysis revealed several potential ubiquitin acceptor sites within the L1 HPV-16 protein. All of them are distributed throughout the entire L1 protein sequence. These sites are conserved between HPV-16 and BPV-1, indicating a probable shared evolutionary function. Moreover, we observed that native BPV-1 virus isolated from bovine papillomas was ubiquitylated at the conserved K452 and K454 residues (Fig. S1). Interestingly, two of the L1 mutated ubiquitin acceptor sites (K64 and K152) are situated on surface loops BC and DE, respectively, which are crucial for capsid formation. The N-terminal part of L1 facilitates interactions during capsid assembly and stabilizes the bonds between L1 monomers. Interestingly, we observed that L1 K64R and K152R mutants were unable to produce progeny PsVs.

It has been shown that viruses can hijack the ubiquitin system, leading to the ubiquitination of their viral proteins to allow their capsid assembly (21). One example is the influenza virus, where a K78R mutation on the M2 protein affects the assembly and production of infectious virus particles (44). However, only one of these HPV L1 mutations (K152R) seems to affect the stability of L1, which may lead to lower PsV production. In addition, we cannot rule out the possibility that these mutations affect the whole virion structure. Whether ubiquitination at these sites is relevant for PsV assembly needs further investigation.

Mutations at other L1 sites, K20R, K217R, K437R, and K452R/K454R, did not affect PsV production or virion stability. However, some of these mutant PsVs were affected in their capacity to infect HaCaT, HeLa, and NIKS cells, although to different degrees. The mutant K452R/K454R was the most defective, with a reduction of more than 50% in all cell lines tested, making it a good candidate for subsequent functional studies, including cell entry, intracellular trafficking, and intracellular processing.

Interestingly, K452 has been shown to be one of the amino acids in L1 responsible for binding to the viral receptor, heparin, and oligosaccharides (45). In addition, it has been reported that the N450A/K452A mutant cannot be stably expressed (45). However, this does not appear to be the case in our study, as we did not observe any significant effects on the assembly or stability of K452R/K454R PsVs or any reduction in viral attachment. Instead, we found changes in viral processing and intracellular trafficking.

Other nearby lysines, K442 and K443, have also been shown previously to be essential for the activation of HPV-16 by heparin. Their mutation (K442A/K443A) impairs the enlargement and softening of the HPV capsid, which may facilitate L1 proteolytic cleavage and subsequent L2 externalization, both of which are important for cell entry (46).

The K452R/K454R mutant is degraded faster over the time of infection. The rescue of L1 by a lysosome inhibitor confirms that the reduced L1 levels result from enhanced lysosomal degradation rather than from delayed internalization. However, it is also possible that the lack of ubiquitin binding to L1 triggers structural changes that alter the plasticity of the human papillomavirus capsid and increase its susceptibility to lysosomal degradation. Interestingly, it has recently been shown that the ubiquitination of HPV16 L2 increases the trafficking of incoming viruses to the lysosome (41).

Intracellular trafficking of the HPV-16 L1 K452R/K454R mutant showed lower colocalization with SNX17-positive vesicles, faster L1 degradation, and higher colocalization with lysosomal compartments, suggesting accelerated and/or aberrant trafficking of L1 ubiquitin-deficient HPV-16 capsids. This may lead to inefficient disassembly of the viral capsids in the time frame that allows normal HPV-16 capsid trafficking and timely rescue of the L2-DNA complexes from the degradational pathway. Ultimately, all these mechanisms together reduce the infectivity of HPV-16 PsVs deficient in L1 ubiquitination.

Although the infectious entry mechanism varies across viral families, numerous viruses use the ubiquitin conjugative system to enhance this process at different stages, such as virus attachment, transport, or uncoating (20). Many viral proteins, such as those from other nonenveloped viruses, for example, adeno-associated viruses (AAV), and the envelope proteins from some flaviviruses, can be ubiquitinated (21, 47). Interestingly, in the case of the Zika virus, ubiquitination of the envelope (E) protein at K38 promotes interaction with the TIM-1 receptor, thereby enhancing virus attachment and entry into cells. In the same line, the recombinant mutant Zika virus EK38R showed a lower infectivity (48). In addition, certain viruses, such as paramyxoviruses, utilize the ubiquitin conjugation system for capsid transport (49–51), while others, including Dengue virus (52), Yellow Fever virus (53), and adenoviruses (54), use ubiquitination to aid in the uncoating process.

While the role of ubiquitin in virus-induced cancer development is relatively well established (22), its involvement in key stages of the viral life cycle, such as entry, intracellular trafficking, assembly, and release, remains largely uninvestigated. It is well-documented that some viral receptors or proteins undergo ubiquitination, which facilitates the recruitment of the endosomal sorting complexes required for transport (ESCRT) machinery. ESCRT components recognize these ubiquitinated proteins and direct them through endosomal pathways, thereby facilitating processes like viral entry (e.g., KSHV) or the budding and release of viruses from infected cells (e.g., HTLV, HBV, and HCV) (55–58). Interestingly, several studies have reported that ESCRT components play an important role during HPV infection (40, 59, 60); however, the specific role of ubiquitin in this context has yet to be clarified.

Future studies will focus on determining whether the ubiquitination of the HPV L1 protein affects its interaction with any specific cellular components. While our results suggest that ubiquitination of L1 could have a potential role during virus intracellular processing, we cannot entirely rule out the possibility that the introduced K>R mutations exert subtle effects on virion structure or L1 protein function. Nevertheless, all our experiments showed that the stability of the L1 mutants remained unaffected: the EM images revealed no apparent structural differences compared with the wild-type HPV-16 PsVs, and neither the ratios of L1 to L2 within the capsid nor the efficiency of pseudogenome incorporation were affected. All this indicates that mutant PsVs are not structurally compromised. Moreover, the use of the ubiquitination inhibitor TAK-243 and specific anti-ubiquitin antibodies during PsV production and infection assays supports a functional role for L1 ubiquitination during HPV infection, rather than indirect effects caused by mutation-induced abnormalities in L1 structure or function. Our work raises several questions: when is L1 ubiquitinated, which specific ubiquitin ligase(s) are involved in the process, and how does L1 ubiquitination regulate virus intracellular processing? Since the C-terminal arm of L1 stabilizes the capsid structure and potentially interacts with host cell factors during viral entry, mutations at these sites could disrupt interactions with certain cellular proteins, thereby inhibiting or delaying the infectious entry process. This hypothesis is supported by the fact that the Ub antibody significantly reduces HPV-16 infection, suggesting that ubiquitin plays an important role in the HPV infectious entry process and that some of the Ub-acceptor sites are exposed on the surface of PsVs. Current studies are focused on answering these questions. In summary, our data show that HPV-16 L1 can undergo ubiquitination and that mutations at these sites affect PsV production and viral infection. These findings could indicate L1 ubiquitination as a potential novel therapeutic target for treating HPV infection.

## MATERIALS AND METHODS

### Cell lines

HeLa, HaCaT, and HEK293TT cell lines (from ATCC) were maintained in DMEM, supplemented with 10% fetal bovine serum, penicillin-streptomycin (100 U/mL), and glutamine (300 mg/mL). The NIKS cells (Normal Immortalized Keratinocytes) were maintained in F medium composed of 75% Ham’s F12 medium and 25% DMEM and supplemented with 5% fetal bovine serum (FBS), adenine (24 μg/mL), cholera toxin (8.4 ng/mL), epidermal growth factor (10 ng/mL), hydrocortisone (2.4 μg/mL), and insulin (5 μg/mL) (61).

### Plasmids

The substitution of arginine for the lysine residues at the L1 Ub-acceptor sites in the context of the pUF3-16L1h and psheLL HPV16 plasmids (used for PsV production) was accomplished using the GeneArt Site-Directed Mutagenesis System (Invitrogen). The mutant plasmids were confirmed by DNA sequencing.

### Antibodies

The following primary antibodies were used in these studies: mouse anti-HPV-16 L1 (CAMVIR-1; Abcam), mouse anti-HPV-16 L2 (2JGmab#5, Santa Cruz Biotechnology), mouse anti-HPV-16E7 (NM2, Santa Cruz Biotechnology), mouse anti-HPV-18E6 (G-7, Santa Cruz Biotechnology), mouse anti-ubiquitin (P4D1, Santa Cruz Biotechnology), mouse anti-alpha-actinin (H-2, Santa Cruz Biotechnology), mouse anti-β-galactosidase (β-Gal) (Promega), mouse anti-LC3 (Sigma), mouse anti-HA (Roche), rabbit anti-SNX17 (HPA043867, Atlas Antibodies), and rabbit anti-LAMP1 (D2D11; Cell Signaling). HPV-16 L1-specific mouse monoclonal antibody 33L1-7 was a kind gift from Martin Sapp (LSU Health Shreveport, USA), while Neil Christensen (Penn State, Cancer Institute, USA) kindly provided the anti-L1 neutralizing antibody (H16.V5). Host-matched secondary antibodies conjugated to horseradish peroxidase (HRP, DAKO), rhodamine, or AlexaFluor dyes 488 and 546 (Molecular Probes) were used as indicated in the text.

### Pseudovirus (PsV) production

PsVs containing a luciferase reporter plasmid (pGL3) were generated in 293TT cells, as described previously (30). Purity and capsid protein content were determined by SDS-PAGE and Coomassie Brilliant Blue staining. The encapsidated DNA was analyzed by real-time PCR, and the copy number was quantified using a standard curve of reporter plasmid DNA.

### Negative staining transmission electron microscopy

Wild-type and mutant (K20R, K64R, K152R, K217R, K437R, and K452R/K454R) HPV PsVs were purified using CsCl gradient and resuspended in HBS buffer (with 0.05% Triton X-100, 0.5% EtOH) at 100 ng; 2 µL of each sample was applied to glow-discharged copper, 400-mesh, formvar-coated grids and incubated for 2–3 min. After a short water wash, grids were stained with 1% uranyl acetate for 10 s, blotted to remove the excess stain, and air-dried. Grids were examined with a Tecnai G2 Spirit BioTwin transmission electron microscope (FEI / Thermo Fisher Scientific), operated at 80 kV using TEM User interface (v. 4.2) and equipped with a LaB6 filament. Images were recorded with a MegaView III G2 CCD camera (EMSIS), using iTEM v.5 software (both Olympus Soft Imaging solutions/EMSIS).

### BPV-1 virion preparation

BPV-1 virions were isolated from cow warts as previously described (28, 62).

### Mass spectrometry analysis

HPV-16 and BPV-1 PsVs produced in cultured cells, and native BPV-1 virions isolated from bovine papillomas were purified by cesium chloride gradient centrifugation and analyzed by mass spectrometry as described previously (62). The samples were supplemented with triethylammonium bicarbonate (pH 8.5) to 20 mM and reduced with 5 mM Tris (2-carboxyethyl) phosphine and heated to 95°C for 5 min. The cysteine residues were alkylated with 10 mM chloroacetamide for 1 h at room temperature to avoid alkylation artifacts (63). After 1 h at room temperature, the samples were digested by the addition of 100 ng trypsin or chymotrypsin in 20 μL of 20 mM triethylammonium bicarbonate (pH 8.5) for 16 h at room temperature. Following digestion, the supernatant was passed 2x over a STAGE tip (64) and eluted with 15 μL of 65% acetonitrile and 0.1% formic acid. The samples were dried and resuspended in 10 μL of 0.1% formic acid and injected onto a 170 mm × 0.075 μm custom-packed columns. The column was custom-packed using Ascentis Express RPA resin (Sigma Aldrich). The column was developed over 60 min using a 0.1% formic acid to 80% acetonitrile gradient. The effluent of the column was sprayed directly into an Amazon ETD mass spectrometer (Bruker Daltonics). Each precursor scan was followed by five fragmentation scans using a dynamic exclusion window of 30 s. The resulting spectra were converted into peak lists using the Data Analysis software (Bruker Daltonics) and analyzed using the X!Tandem search engine (65). The analysis included the complete modification of Cys (+57.02) and variable modifications for oxidation of Met (+15.9), deamidation of Asn and Gln (+0.98), phosphorylation of Ser, Thr, or Tyr (+79.9), and the Ubiquitin mark on Lys (+114.04) (66). The resulting spectral matches were filtered with a false discovery rate of < 1%. Modification sites were manually curated to verify the correct localization of the modifications.

### Ubiquitination assays

The bicistronic plasmids expressing HPV-16 L1 and L2 were transfected into HEK293TT cells, together with a luciferase reporter and HA-Ub or pcDNA plasmids. After 48 h, the cells were harvested, and pseudovirions were purified by cesium chloride gradient. Purified PsVs+HA Ub and PsVs+pcDNA were immunoprecipitated using HA agarose beads (Sigma) and analyzed by western blotting using the anti-HPV-16 L1 antibody.

HEK293 cells were transfected with L1 wild-type plasmid together with pcDNA or HA-ubiquitin. After 24 h, the cells were lysed with lysis buffer (50 mM HEPES [pH 7.4], 150 mM NaCl, 1 mM MgCl2, and 1% Triton X-100) supplemented with a protease inhibitor cocktail (PIC; Calbiochem). Lysates were divided, and half was immunoprecipitated using anti-HA antibody-conjugated agarose beads, while the second half was incubated with an anti-L1 antibody. The ubiquitinated L1 was then detected by western blotting using an anti-HPV-16 L1 antibody or an anti-HA antibody, respectively.

Next, HEK293 cells were transfected with L1 wild-type or mutants (K20R, K64R, K152R, K217R, K437R, and K452R/K454R) plasmids together with pcDNA or HA-ubiquitin. After 24 h, cells were lysed with lysis buffer (as above) supplemented with PIC (Calbiochem), and incubated with anti-HPV-16 L1 antibody overnight on a rotating wheel at 4°C. The immunocomplexes were incubated with protein G Sepharose (Amersham Biosciences) for 3 h at 4°C. After several washes, ubiquitination of the immunoprecipitated L1 protein was analyzed by western blotting.

### Analysis of L1 expression level

HEK293TT cells were transfected with plasmids expressing the wild-type and the mutated L1 (K20R, K64R, K152R, K217R, K437R, and K452R/K454R), and 100 ng of lacZ plasmid to control for transfection efficiency. After 24 h, cells were incubated with or without proteasome inhibitor CBZ (MG132 Z-Leu-Leu-Leu-al; Sigma-Aldrich no. C2211), harvested 24 h later, and analyzed by western blotting, using anti-HPV-16 L1 and anti-β-galactosidase antibodies. β-Gal was used as a loading and transfection efficiency control. As a CBZ treatment-positive control, cells were transfected with plasmids expressing HPV-16 E7 and analyzed by western blotting with an anti-HPV-16 E7 antibody.

HEK293TT cells were transfected with psheLL plasmids expressing HPV 16 L1 and L2, containing the wild-type or the mutant L1, as indicated, and 100 ng of lacZ plasmid as a control for transfection efficiency. After 24 h, DMSO or the proteasome inhibitor CBZ was added, and cells were harvested a further 24 h later. Cell lysates were collected and analyzed by western blotting, using anti-HPV-16 L1, anti-HPV-16 L2, and anti-β-galactosidase antibodies.

HEK293TT cells were transfected with plasmids expressing the wild-type or the respective mutated L1, and 100 ng of lacZ plasmid as a control for transfection efficiency. After 24 h, the cells were incubated with or without chloroquine and harvested 24 h later. Cell lysates were collected and analyzed by western blotting, using anti-HPV-16-L1, anti-β-galactosidase antibody, and anti-LC3 antibody as a chloroquine treatment-positive control.

### Non-reducing denaturing gel analysis

The pattern of disulfide bonds in the PsV capsids was examined as previously described (29, 62). HPV-16 pseudovirion preparations were alkylated using N-ethyl maleimide (NEM, Sigma). Alkylation was conducted by diluting roughly 1 µg of pseudovirion preparations into 10 mM NaPO_4_ (pH 6.5) and then adding 10 mM NEM for 10 min at room temperature. Samples were next mixed with an equal volume of non-reducing SDS-PAGE loading buffer (2% SDS, 100 mM Tris pH 6.8, 10 mM NEM, 5 mM EDTA, 10% glycerol, and 0.01% bromophenol blue) and incubated at room temperature for 10 min and then at 65°C for 10 min, prior to electrophoresis on 7.5% SDS polyacrylamide gels. Samples were then transferred to a nitrocellulose membrane and stained using Ponceau S solution (Sigma).

### Trypsin digestion of capsids

The sensitivity of the PsVs to trypsin digestion was determined as previously described (31, 62). Briefly, 10 μL PsVs (500 ng) was incubated with 10 μL of 0.05% trypsin at 37°C for either 30 min or 3 h. Following the digestion period, 4 μL of the PsV mixture was processed for L1 detection, and 16 μL was processed for L2 detection. Samples were resolved on 10% SDS-PAGE gel, followed by western blotting; L1 was detected with CAMVIR-1 and L2 with mouse anti-16 L2 (16.D4 64-81) antibodies.

### Infectivity assays

HaCaT, HeLa, and NIKS cells were seeded in 12-well plates at a density of 0.5 × 10^5^ cells/well. After 24 h, cells were exposed to 100 vge/cell of wild-type and mutated luciferase reporter-positive PsVs. Infection was monitored after 48 h by luminometric analysis of firefly luciferase activity, using the Luciferase Assay System (Promega). The infection efficiency of mutated PsVs was calculated by normalizing the values with wild-type PsVs. Equal amounts of total cell protein extract were used in the luciferase measurements. Cells were analyzed by one-way ANOVA with Dunnett’s multiple comparison test, comparing wild-type and mutant HPV 16 PsVs. Multiplicity-adjusted *P* values were calculated for each comparison.

### Ubiquitin antibody assay

HPV-16 wild-type PsVs were incubated with increased concentration (1:1,000, 1:250, 1:100) of anti-HPV-18 E6 as a control (G-7, Santa Cruz Biotechnology, concentration 200 µg/mL), anti-L1 (H16.V5) neutralizing (note that original stock was first diluted in PBS to a concentration of 1:500) (15), and anti-ubiquitin antibodies (P4D1 and A-5 Santa Cruz Biotechnology, concentration 200 µg/mL) for 2h at 37°C and then used to infect HaCaT cells at 100 vge/cell. After 48 h, the cells were harvested, and the levels of luciferase activity were measured in triplicate by luminometry. Cells were analyzed by one-way ANOVA with Dunnett’s multiple comparison test, comparing non-treated and antibody-treated wild-type HPV-16 PsVs. Multiplicity-adjusted *P* values were calculated for each comparison.

### Cell viability assay

Cell viability following treatment with increasing concentrations of the TAK-243 inhibitor (MLN7243; Selleckchem, Houston, USA) was assessed using the PreMix WST-1 Cell Proliferation Assay System (TaKaRa, San Jose, CA, USA). HEK293TT cells were incubated for 48 h with TAK-243 at concentrations of 10 nM, 50 nM, 100 nM, 500 nM, and 1,000 nM, with DMSO as the vehicle control. Subsequently, PreMix WST-1 reagent was added, and cells were incubated for 1 h at 37°C in a 5% CO₂ atmosphere. Absorbance was measured at two wavelengths (450 nm for signal detection and 620 nm as a reference) using a Synergy H1 microplate reader (BioTek).

### Viral binding and entry assays

HaCaT cells were chilled on ice for 20 min before infecting with 1 μg L1/mL of wild-type and K452R/K454R HPV-16 PsVs. Plates were then incubated on ice for 1 h to allow surface binding of the PsV particle. The control groups were then washed with cold high-pH PBS (pH = 10.75) to remove the surface-bound virus. Samples were collected and resolved on 10% SDS-PAGE gels, followed by western blotting; L1 was detected with CAMVIR-1 antibody. For the entry assay, after a 1-h PsV pre-binding step on ice, cells were washed with cold PBS (pH = 7.4) to remove unbound PsV particles, re-fed with fresh media, and incubated for the indicated times at 37°C, 5% CO2. After 1, 2, and 4 h, cells were washed with high-pH PBS to remove the surface-bound virus. The assay was repeated in the presence of ammonium chloride. Samples were then collected and resolved on 10% SDS-PAGE gel, followed by western blot as described above. Band intensities of L1 were analyzed using ImageJ software, and the amount of L1 was normalized to the GAPDH loading control. Cells were analyzed by two-way ANOVA with Šidak’s multiple comparisons test, comparing wild-type and mutant HPV-16 PsVs at different time points. Multiplicity-adjusted *P* values were calculated for each comparison.

### Assessment of PsV internalization by flow cytometry

HaCaT cells were rapidly pre-chilled on ice and then infected with 1 μg L1/mL wild-type and K452R/K454R HPV-16 PsVs conjugated with AF488. After a 1-h incubation on ice to allow surface binding of virions, the control samples were harvested by trypsinization and immediately analyzed by flow cytometry. For internalization, cells were washed with cold PBS (pH = 7.4) to remove unbound virions and incubated in fresh medium at 37°C and 5% CO_2_ for 45 min before trypsinization and flow cytometry. Analysis was performed on a Guava easyCyte (Luminex) together with Guava InCyte software. Quadrant statistics from three independent experiments in duplicate were used to calculate the means ± SD of AF488-positive cells, with a minimum of 3,000 cells per measurement. Internalization of wild-type and K452R/K454R HPV-16 PsVs was statistically analyzed by an unpaired *t*-test (two samples).

### Immunofluorescence and confocal microscopy

HaCaT cells were seeded on 9-mm sterile coverslips at a density of 0.5 × 10^5^ cells per well in 12-well plates and grown overnight at 37°C. For attachment and intracellular trafficking experiments, 1 μg L1/mL wild-type and K452R/K454R HPV-16 PsVs were added to the cells and allowed to bind for 1 h at 4°C. Cells were then rinsed with cold PBS and either immediately fixed with 4% (vol/vol) paraformaldehyde in PBS for 15 min at room temperature (attachment) or incubated in fresh medium at 37°C and 5% CO_2_ for the indicated time points (trafficking). For the attachment analysis, non-permeabilized cells were counterstained with 4,6-diamidino-2-phenylindole (DAPI) (Sigma), and mounted on glass slides immediately after attachment at 4°C. For the internalization study, cells were washed and fixed with 4% (vol/vol) paraformaldehyde in PBS at the indicated time intervals, followed by permeabilization in PBS/0.1% Triton at room temperature for 5 min. The cells were then incubated with primary antibodies at 37°C for 1 h. After washing, the cells were incubated with AF488- or AF546-conjugated secondary antibodies (Molecular Probes) for 30 min at 37°C, followed by another extensive wash in PBS. The cells were then counterstained with DAPI, washed, and mounted on glass slides. Images of the cells were acquired using an Observer.Z1 microscope (Zeiss) attached to an LSM 710 confocal unit and ZEN software. Colocalization analysis was performed using ImageJ software (67) with the JACoP plug-in (68). The percentages of PsVs colocalizing with SNX17 or LAMP1 were determined using Mander’s Colocalization coefficients for two channels. To quantify the L1 signal upon L1 uncoating and total L1, cells (ROIs) were analyzed for the intensity of the L1 uncoating signal (33L1-7) and presented as corrected total cell fluorescence (CTCF) per cell normalized to the amount of total L1 signal. Internalization and colocalization data of wild-type and K452R/K454R HPV-16 PsVs at a given time point were statistically analyzed by unpaired *t*-test (two samples).

### Statistics

All experiments were performed at least three times. Statistical analyses were performed using GraphPad Prism 10 software (GraphPad Software, CA, USA). GraphPad Prism 10 was also used for data calculation, tabulation, and graphical representations. The results of the statistical analyses were considered significant at *P* < 0.05.

## Data Availability

Data will be made available on request.

## SUPPLEMENTAL MATERIAL

Figure S1 jvi.00285-26-s0001.doc Identification of conserved K452 and K454 ubiquitin acceptor sites in the BPV-1 L1 capsid protein.

ASM does not own the copyrights to Supplemental Material that may be linked to, or accessed through, an article. The authors have granted ASM a non-exclusive, world-wide license to publish the Supplemental Material files. Please contact the corresponding author directly for reuse.
