# Supplementary material for: Regulation of HPV-16 infection by ubiquitination of the L1 major capsid protein
Source: J Virol. 2026 Apr 13;100(5):e00285-26. doi: 10.1128/jvi.00285-26 (PMC13185570; doi:10.1128/jvi.00285-26)
Supplement: Figure S1 — Identification of conserved K452 and K454 ubiquitin acceptor sites in the BPV-1 L1 capsid protein. [file jvi.00285-26-s0001.doc]

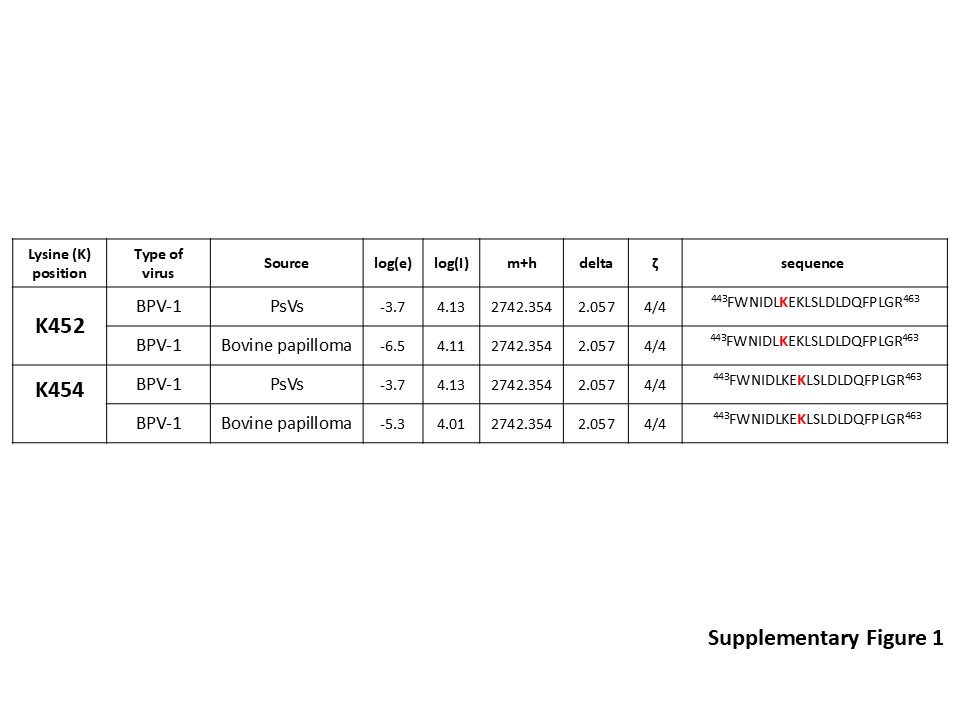


**Supplementary Figure 1.** Identification of conserved K452 and K454 ubiquitin acceptor sites in the BPV-1 L1 capsid protein. Conserved ubiquitin (Ub) acceptor lysines K452 and K454 were identified in the BPV-1 L1 capsid protein from BPV-1 pseudovirions (PsVs) produced in cultured cells and from native BPV particles purified from bovine papillomas by mass spectrometry. The table summarizes the mass spectrometric analysis: log(e) is the base 10 log of the expectation that the assignment is stochastic; log(l) is the base 10 log of the sum of the intensities of the fragment ion spectra; m+h is the calculated mass of the protonated parent ion for this sequence assignment; delta is the difference between the measured and calculated protonated parent ion masses; ζ is the ratio of the measured charge of the parent ion to the number of basic sites in the assigned peptide sequence. The sequence of the assigned peptide is also shown.
